# Modeling the Salinity Effect on the Water Retention Curve of Geosynthetic Clay Liner (GCL) on the Drying Path

**DOI:** 10.3390/ma16155468

**Published:** 2023-08-04

**Authors:** Zhenming Zeng, Yi Lu, Tong Wan, Shan Lin, Xingzhong Nong, Jiajun Sun

**Affiliations:** 1 School of Civil Engineering, Guangzhou University, Guangzhou 510623, China; zhenmingzeng@outlook.com (Z.Z.); 2112116009@e.gzhu.edu.cn (T.W.); 2112116215@e.gzhu.edu.cn (J.S.); 2 Guangzhou Metro Design & Research Institute Co., Ltd., Guangzhou 510010, China; linshan@dtsjy.com (S.L.); nongxingzhong@gmdi.cn (X.N.)

**Keywords:** geosynthetic clay liners (GCLs), water retention curve (WRC), salinity, matric suction

## Abstract

The water retention curve (WRC) of a geosynthetic clay liner (GCL) is influenced by the presence of exchangeable cations in the leachate during changes in water content in a landfill construction. This research aims to investigate the impact of salinity on the WRC of GCL. To measure the WRC of GCL under different sodium chloride (NaCl) concentrations on the drying path, a chilled-mirror dew-point device capable of controlling the GCL’s volume was employed. Additionally, the dry state microstructure of the GCL was examined using electron microscopy. The test outcomes indicate that GCL hydrated with higher salinity has greater suction at the same water content during drying. This influence can be attributed to changes in salinity and the precipitation of NaCl crystals within the bentonite when water evaporates, which in turn affects the bentonite’s microstructure and leads to increased matric suction. By introducing the Fredlund and Xing model and parameter relationship, it is possible to predict the WRC of GCL under salinity effects after measuring the WRC under different salinity conditions on the drying path.

## 1. Introduction

Geosynthetic clay liners (GCLs) are commonly employed as anti-seepage barriers in landfills to prevent the migration of pollutants from landfill sites into the surrounding underground environment. GCLs typically comprise a layer of bentonite sandwiched between two geotextiles, which are joined together through needling, bonding, or sewing techniques. This construction effectively restricts the outward movement of contaminants, mitigating the potential pollution of subsurface areas [1,2,3]. The engineering performance needs to be provided by the bentonite after hydration and mainly controlled by its water saturation level [1,4,5]. The performance of GCLs is very sensitive to environmental conditions of bentonite, such as monovalent or divalent salts dissolved by fluid species [6,7], the desaturation caused by dehydration due to changes in groundwater level or leachate [8,9], and the impact caused by an aggressive pH value [10]. The change in partial saturation and WRC under the influence of saline solutions (such as CaCl_2_ and NaCl) has been reported [11,12]. Therefore, it is imperative to assess the efficacy of the GCL as an anti-seepage barrier across various water content conditions. A comprehensive understanding of the water absorption and dehydration characteristics of GCLs with a constant volume is crucial for their long-term performance evaluation [13,14,15]. The water retention curve (WRC) describes the relationship between water content and suction in unsaturated soils. The WRC can be used to estimate the properties of soil under unsaturated conditions including permeability, volume change, deformability, and shear strength [16,17,18,19]. The existing literature shows that the influence of density and salt solution concentration has been considered in the study of WRC, and the concentration of porous fluid has a significant influence on the shape of WRC, but most studies of the water retention performance of GCLs are limited to the wetting path [1,20,21,22]. However, when studying the WRC of GCLs hydrated by salt solution on the drying path, the concentration of pore solution is constantly changing. Different from the bentonite, the GCL can be obtained by treating it as a composite material; that is, evaluating it as a whole, which is more conducive to the WRC of GCL performance evaluation.

GCLs are frequently exposed to leachate in landfills, which often contains high levels of salts derived from waste materials [4,5,23] and a substantial quantity of exchangeable cations. Alterations in pore water chemistry from ground water infiltration can impact the microstructure of the compacted bentonite owing to cation exchange and changes in diffuse double layer thickness [24,25]. The presence of these cations significantly influences the water retention behavior of GCLs, consequently affecting their overall performance and effectiveness as barrier systems [17,21,23]. The hydration electric double layer refers to the interface between the solid particles of the GCL and the surrounding fluid, which is influenced by the presence of charged ions. When leachate interacts with the GCL, the exchangeable cations in the leachate migrate to the GCL’s surface, leading to changes in the electric double layer. These changes, in turn, impact the function of the GCL to retain water and control its movement [4,7,15,17,21]. Understanding the water retention performance of GCLs under various salt concentrations is crucial to evaluating their long-term serviceability. The water retention properties of GCLs directly influence their function to prevent the migration of contaminants and pollutants from landfills into the surrounding environment [1,2]. Therefore, comprehensive research is necessary to investigate the effect of different salt concentrations on the water retention behavior of GCLs. While there is existing literature on GCLs in nonstandard solutions, much of the focus has been on permeability aspects [23,26,27]. However, it is essential to expand research efforts to encompass a broader understanding of the impact of high salt content leachate on the water retention capabilities of GCLs. By investigating and characterizing the behavior of GCLs under these conditions, engineers and researchers can enhance their design and performance in landfill applications, ultimately contributing to more effective and environmentally responsible waste management practices.

This research aims to comprehensively investigate the WRC of GCLs with a constant volume in the presence of salt solution, specifically sodium chloride (NaCl). In this study, the focus extends beyond the WRC itself to examine the impact of NaCl solution and NaCl crystal formation along the drying path of GCLs. Scanning electron microscopy (SEM) analysis will be employed to visualize and analyze the microstructure of GCLs under different salt concentrations. This microscopic examination will shed light on how the presence of NaCl solution and the subsequent precipitation of NaCl crystals affect the internal structure and pore distribution of GCLs. To predict the influence of the NaCl factor on the WRC of GCLs in the drying path, the well-established Fredlund and Xing model will be utilized. By combining the empirical relationships derived from the WRC measurements in distilled water (DW) with the experimental data obtained under salt solution conditions, a comprehensive understanding of the impact of NaCl on the water retention behavior of GCLs will be achieved. This research not only enhances our knowledge of GCL behavior in the presence of salinity, but also contributes to the development of reliable models for predicting their performance under varying environmental conditions. Ultimately, these findings will facilitate a more accurate design and evaluation of GCL systems, enabling a more effective management of landfills and minimizing the potential environmental risks associated with leachate migration.

## 2. Materials and Methods

Table 1 presents the engineering properties of the sodium-based GCL used in this study. Laboratory testing yielded the following results for the GCL specimen: the mass per unit area ranges from 4138 to 4452 g/m^2^, the thickness measures between 5.8 and 6.4 mm under the stress of 2 kPa, and the natural gravimetric water content varies from 6.56% to 9.01%.

In the experimental setup, the injected liquids include distilled water (DW) and various sodium chloride (NaCl) solutions with concentrations of 0.1 M, 0.2 M, 0.5 M, and 2 M. These detailed properties and experimental conditions lay the foundation for the subsequent analysis and evaluation of the water retention behavior of the sodium-based GCL. The varying NaCl concentrations provide insights into the influence of salinity on the GCL’s water retention characteristics, enabling a comprehensive understanding of its performance under different salt solution conditions.

In this study, the WP4C dew point water potential meter produced by Decagon Company was adopted Under the condition of free expansion, the internal limiting pressure generated by the geotextile in GCL will have different suction values, even if the WRC measured under the condition of constant expansion and free expansion is small [14]. 

The main test steps are as follows: an experimental sample with a diameter of 38 mm is gently placed into the sample cup of the WP4C instrument, and the perforated cover is carefully screwed onto the GCL sample. The solution is injected into the GCL sample at 25 °C until it reaches a gravimetric water content of 75%. The sample is then sealed and stored under controlled conditions. During the subsequent drying phase, the GCL sample is placed in a constant temperature and humidity box until the water content of the sample reaches the desired target value. Then, the water content and suction of GCL are measured to provide valuable data of its water retention performance.

Simultaneously, the influence of brine concentration on the microstructure of the bentonite within the GCL sample is assessed using a TM3030 Hitachi desktop scanning electron microscope (SEM).

This study aims to gain insights into the water retention characteristics of the GCL sample and the corresponding modifications in its microstructure induced by varying concentrations. This comprehensive approach provides a deeper understanding of the GCL’s behavior and aids in optimizing its performance for diverse engineering applications.

## 3. Results and Discussion

### 3.1. WRC on the Drying Path of GCL

**Figure 1:**
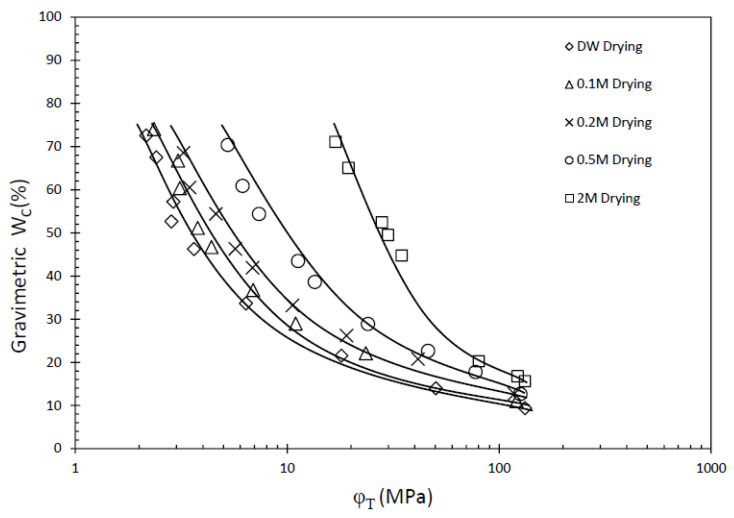
WRC of GCLs in distilled water (DW) and NaCl solution on drying path.

The WRC displayed in Figure 1 shows the relationship between the GCL at varying water salinity conditions during the drying process while maintaining a constant volume. The WRC provides crucial insights into the GCL’s function to retain water under different environmental conditions [4,13]. The results obtained from this study offer valuable observations. It is evident that the water content of the GCL gradually decreases as the total suction of the material increases. Moreover, with increasing salinity, the WRC exhibits a discernible shift towards higher suction values. Conversely, as the water content decreases, the total suction also experiences an upward trend. This suggests that the higher salinity of the hydrated GCL leads to an increase in the suction. Similar results were also found by other authors for both the wetting and drying path [7,9,10].

This implies that both the salinity of the water and water content play significant roles in determining the suction characteristics of the GCL. It is important to note that the total suction ($\varphi_{T}$), as described in Equation (1), comprises two distinct components: matric suction ($\varphi_{M}$) and osmotic suction ($\varphi_{O}$) [28]. These components collectively contribute to the overall suction capacity of the GCL and are instrumental in understanding its water retention behavior.

(1) $$\varphi_{T}=\varphi_{M}+\varphi_{O}$$

Matric suction reflects the water absorption potential of soil particles while osmotic suction reflects the ion content in pore fluid. For GCLs hydrated by water salinity, the osmotic suction combines from the ions on the clay particles dissolved in pore water and the ions in the salinity of hydration water. Therefore, as the salinity of GCL hydration increases, so does the initial total suction of its WRC on the drying path.

**Figure 2:**
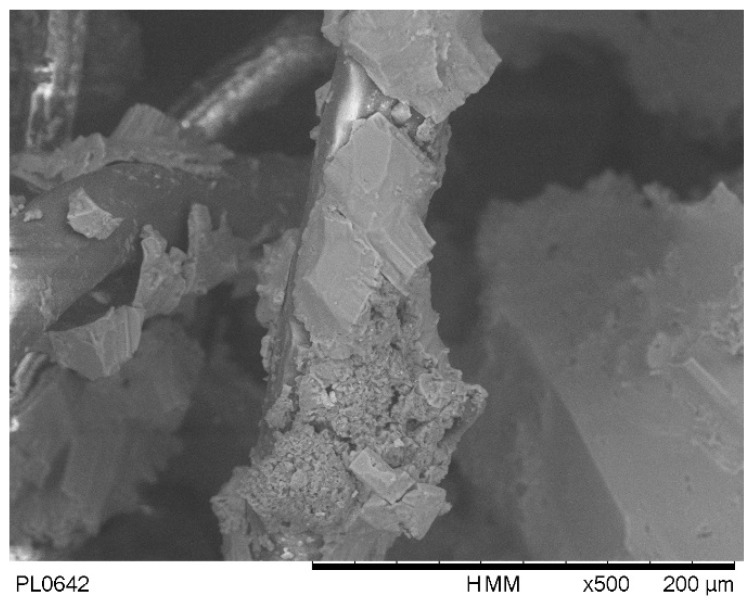
NaCl crystals on fiber of geotextile hydrated by 2 M NaCl solution.

**Figure 3:**
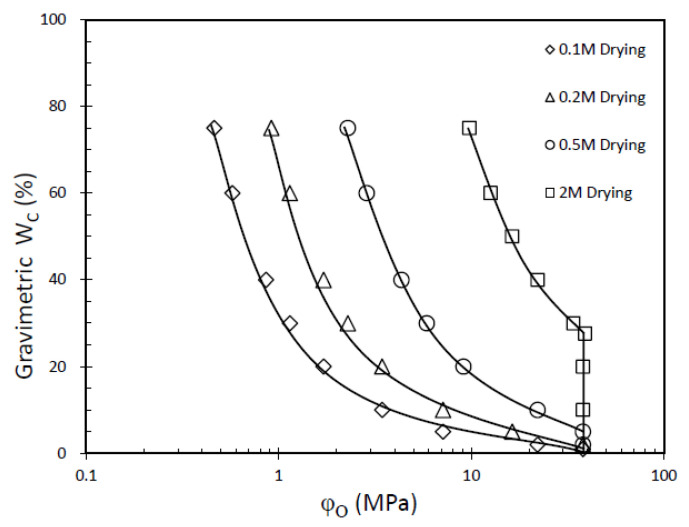
Osmotic suction of GCL with different initial salinity varying with water content.

Through the hydration process of the GCL, sodium chloride (NaCl) ions are consistently drawn into the pores of the sample. Notably, the salt content of the sample remains unchanged after hydration. As the drying process ensues, the water content within the sample diminishes, leading to an increase in the salinity of the pore water. This increase continues until the pore water reaches saturation, at which point NaCl crystals precipitate out of the solution. Figure 2 visually presents the NaCl crystals observed on the fibers of the geotextile after being hydrated with a 2 M NaCl solution. Because of the existence of salt solution, the pore structure of bentonite in GCL will change, and the higher the salinity, the more obvious the pore change [15,16]. Consequently, during the drying process, the salinity of the water within the sample continuously rises, contributing to the continuous increase in the osmotic suction of the pore water. This osmotic suction value steadily increases until it reaches its maximum value when the pore water becomes fully saturated. Figure 3 illustrates this relationship, showcasing the variation in the osmotic suction value as the water salinity increases. The continuous rise in water salinity and corresponding increase in osmotic suction during drying have significant implications for the behavior and performance of the GCL. Understanding these dynamics is crucial to accurately assessing the water retention capabilities and overall effectiveness of GCLs under various conditions. Additionally, the observation of NaCl crystal precipitation highlights the complex interplay between salt concentration, pore water salinity, and the microstructure of the GCL. This knowledge aids in improving the design and performance of GCLs in practical engineering applications.

Without considering the osmotic suction generated by ions dissolved in the pore water during the hydration of clay particles, we assume that the matric suction of GCL samples hydrated by salinity water is equal to the total suction under the condition of DW, and the osmotic suction is equal to the osmotic pressure of the corresponding NaCl solution. Then, the calculation of the total suction for GCLs hydrated by different salinity water can be obtained by adding the corresponding NaCl solution osmotic pressure to the total suction under DW conditions. However, this simple assumption ignores the effect of the existence of NaCl solution on the pores of bentonite, which will lead to the differential value between the total suction that is calculated and measured. However, the existing literature shows that the existence of salt solution will definitely make the matrix suction increase [9,15,16,20]. Since the matric suction under the condition of NaCl solution was not directly measured in this study, in order to explore the effect of salinity on the WRC of the GCL on the drying path other than the osmotic suction, the difference ∆*φ* is obtained by subtracting the calculated total suction value from the measured total suction value of the GCL hydrated by different NaCl solutions for analysis (Figure 3). It is important to recognize that the differential value (∆*φ*) provides insights into the influence of salinity on the WRC of the GCL, highlighting the discrepancies between the calculated and measured total suction values. By considering the impact of NaCl solutions on the pores of the bentonite material, a more comprehensive understanding of the complex behavior of the GCL in response to varying salinity conditions can be attained. This analysis contributes to enhancing the accuracy and reliability of future GCL designs and applications in relation to water retention performance.

**Figure 4:**
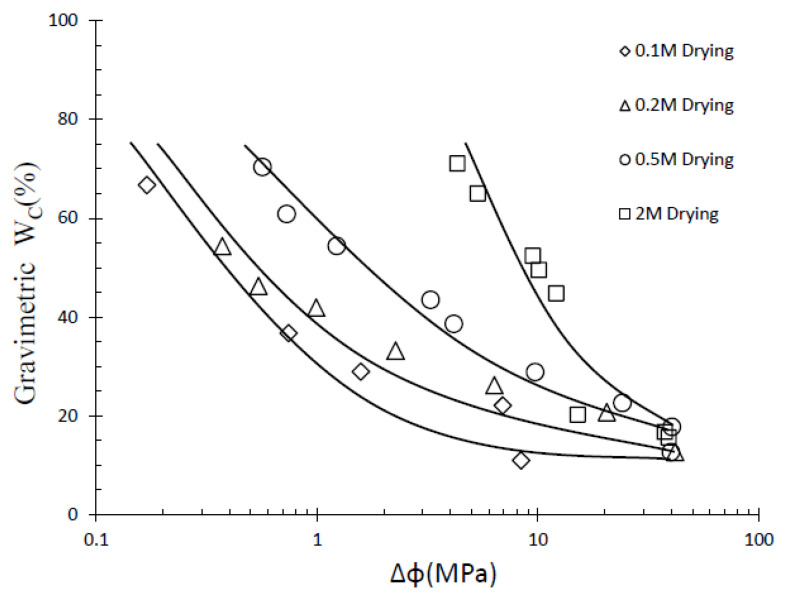
Difference between measured total suction and calculated total suction.

**Figure 5:**
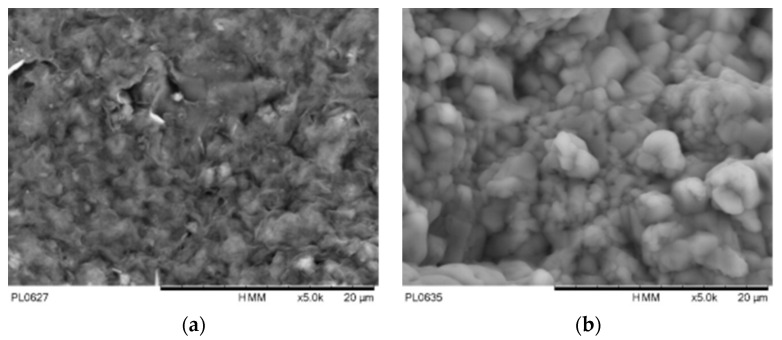
Pore structure of bentonite in DW (**a**) and bentonite 2 M NaCl solution (**b**).

As depicted in Figure 4, the ∆*φ* exhibits an increasing trend with higher GCL hydration salinity and lower gravimetric water content. This observation indicates that the change in ∆*φ* cannot be solely attributed to the osmotic potential resulting from increased salinity. Notably, the curves representing different initial salinities converge towards a similar level as the suction increases. This finding suggests that the influence of salinity on GCL suction encompasses not only osmotic suction, but also the impact on matric suction. According to the principles of diffuse double layer theory, the concentration of cations in the pore solution is inversely correlated with the surface distance between the clay particles. The evaporation of water leads to the increase in NaCl concentration in the solution; that is, the salinity of the pore water in clay increases, and the pore spaces within the bentonite expand [10]. Additionally, existing research has established a direct relationship between ion concentration changes in pore water solution and the microstructure of bentonite. This alteration in the microstructure of the GCL affects its matric suction, thereby causing corresponding changes in the total suction value [19,25]. The scanning electron microscopy (SEM) image depicted in Figure 5 reveals that the bentonite particles within the GCL undergo a transition from a dispersed structure to an agglomerated structure in response to salinity. Notably, during the drying process of the GCL hydrated with a 2 M solution, the precipitation of crystals coincides with a constant osmotic suction. However, the rate of increase in ∆*φ* is accelerated, suggesting that the crystallization within the pore structure may have an impact on the total suction value. This comprehensive analysis emphasizes the multifaceted influence of salinity on the behavior of the GCL, particularly with regard to its water retention characteristics. The findings highlight the interplay between salt concentration, pore structure, and microstructural changes within the GCL. By considering these complex dynamics, a more accurate understanding of the total suction response of the GCL under different salinity conditions can be obtained. These insights contribute to advancing the design and performance evaluation of GCLs, ensuring their effective function as barriers against water infiltration and seepage.

### 3.2. WRC Model Considering Salinity Effect

**Figure 6:**
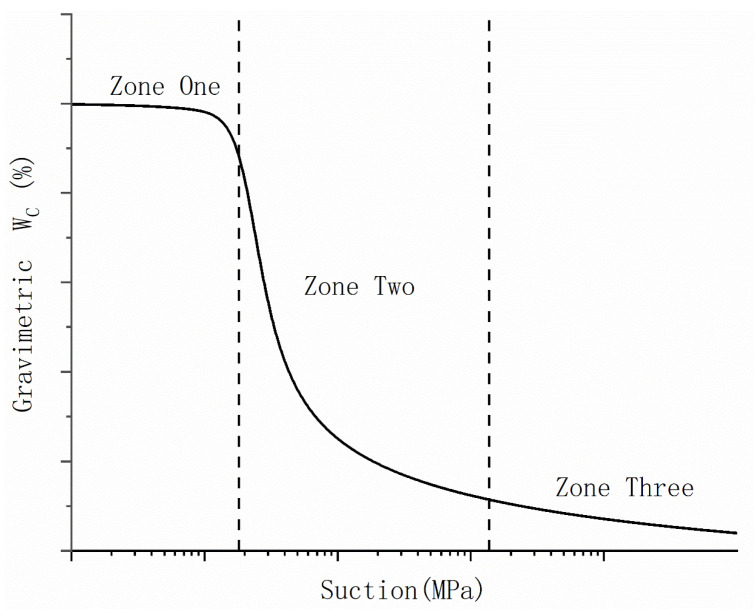
Conceptual model of WRC.

Based on the previous discussion, it is evident that salinity has a significant impact on the drying behavior of GCLs. Consequently, it is crucial to consider the influence of salinity on the WRC of GCLs during the drying process. However, directly measuring the WRC properties of GCLs affected by salinity in engineering applications is challenging. Developing a predictive model that can estimate the WRC of GCLs under the influence of salinity would be beneficial for detecting and studying the hydraulic behavior of GCLs in real working conditions. The conceptual model for the WRC can be derived from the existing literature on unsaturated soil research [29,30,31,32,33]. Figure 6 illustrates the division of the WRC into three stages based on changes in the curve’s slope: high, medium, and low water content. In Zone One, which corresponds to high water content, the salinity in the GCL sample remains low, and there is no significant increase or crystallization of pore water salinity. In Zone Three, the pore water salinity reaches its maximum as the water content decreases. This results in a rapid increase in the total suction, with the matric suction far exceeding the osmotic suction. In Zone Two, as the water content decreases, the pore water salinity in the GCL gradually rises towards saturation, leading to the continuous precipitation of crystals. Consequently, the WRC of the drying path of GCLs in this zone differs from that of pure water. Therefore, the main focus of this experiment is to examine the changes in the WRC within Zone Two.

**Figure 7:**
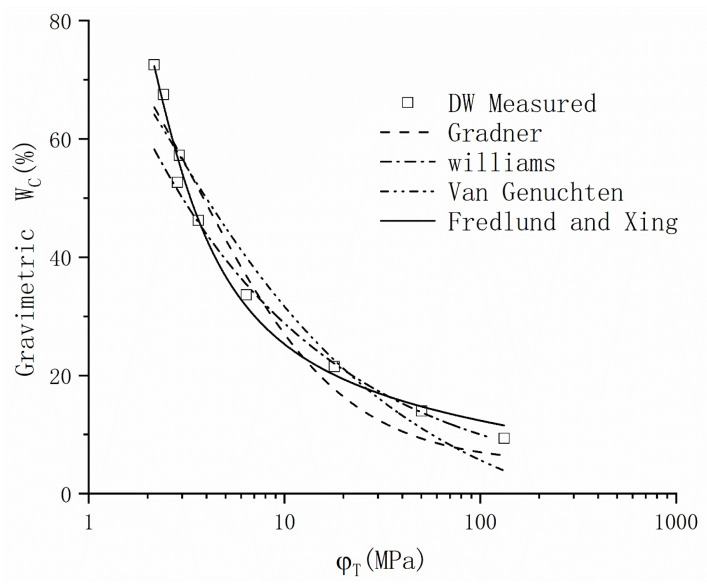
WRC of GCL fitted by classical models.

**Figure 8:**
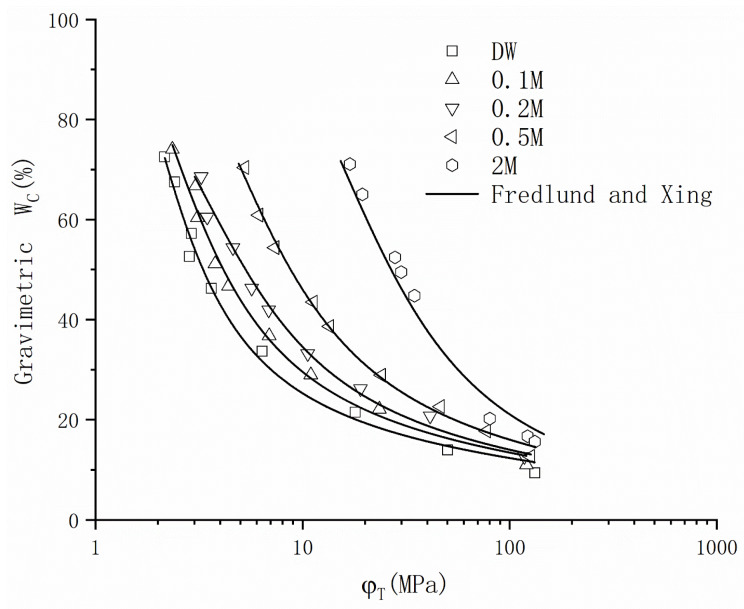
WRC fitted by Fredlund and Xing model.

Table 2 presents a comprehensive overview of the classical models documented in the existing literature. A comparison of the data under DW conditions is conducted to identify an appropriate model. Figure 7 depicts the logarithmic representation of Model 1, which describes an exponential correlation between suction and exhibits with water content. This model demonstrates a satisfactory fit within Zone Two, yet the fitting curve across the entire suction range deviates from the conceptual model. Models 2 to 4 are widely adopted by researchers as classic models, and the physical interpretations of their fitting parameters have been extensively investigated and determined (shown as Table 2). When comparing the fitting outcomes for the WRC within Zone Two, it becomes evident that Model 4 provides a superior fit. Hence, Model 4, also known as the Fredlund and Xing model, is chosen to fit the drying path data of GCL. Figure 8 illustrates the measured GCL data and the fitted lines obtained using the Fredlund and Xing model. The fitting results reveal that, as salinity increases, the curve gradually shifts towards the upper right quadrant. It is noteworthy that the suction value at the initial state of the curve is influenced by the corresponding chemical suction value under the given salinity conditions.

**Figure 9:**
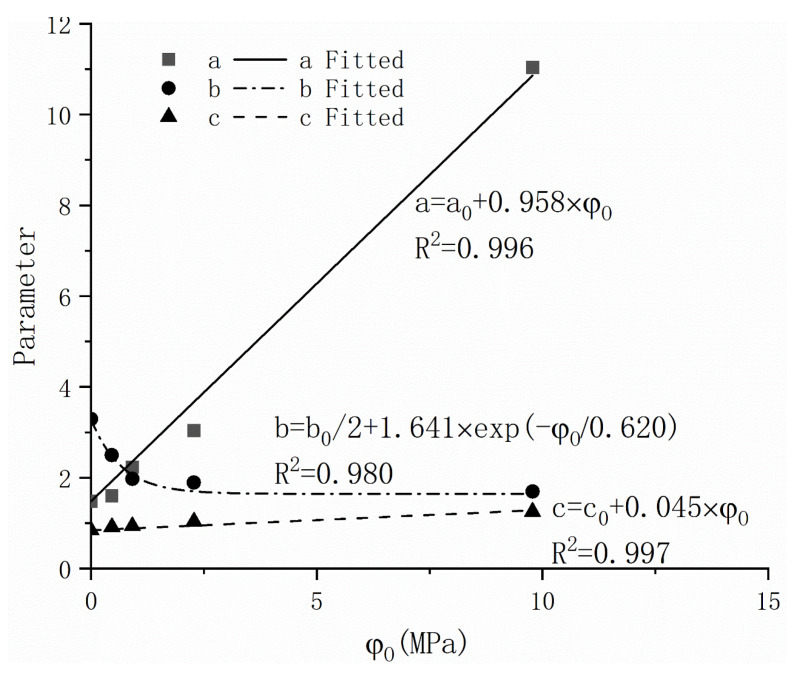
Relationship between parameters a, b, and c and osmotic suction value of salinity.

According to the fitting results of the model, the constants A, B, and C are obtained by the Levenberg arquardt optimization algorithm (Table 3). Then, the relationship between the parameters and experimental influencing factors is studied, and the relationship between coefficients a and b and c at a higher R^2^ value is determined, as shown in Figure 9, and the equation expression is summarized as follows:

(2) $$a=a_{0}+0.958\times \varphi_{O}$$

(3) $$b=\frac{b_{0}}{2}+1.641\times exp(\frac{-\varphi_{O}}{0.62})$$

(4) $$c=c_{0}+0.045\times \varphi_{O}$$

where $\;a_{0}$, $b_{0}$, and $c_{0}$ are the fitting parameters’ value of a, b, and c in DW, and $\varphi_{O}$ is the osmotic suction value of corresponding salinity.

Equation (2) presents an approximate linear relationship between parameter ‘a’, which is related to the air entry value, and the osmotic suction for GCL in the drying path. Similar findings were reported by Karagunduz et al. [34]. Additionally, the results indicate that parameter ‘c’, which is associated with the residual water content, exhibits an approximate linear relationship with the osmotic suction value. Therefore, Equation (4) is employed to represent this relationship. Parameter ‘b’ is determined by the pore size distribution of the sample, which directly influences the slope of WRC in the desaturated region [35]. Previous studies have shown that salinity has an impact on the pore concentration of samples [11,36], consequently affecting the value of ‘b’ and the shape of the WRC. Based on these findings, Equation (3) is proposed to describe the relationship between the value of parameter ‘b’ and salinity (or the corresponding osmotic suction value). Importantly, it should be noted that the values of ‘a_0_’, ‘b_0_’, and ‘c_0_’ are introduced into Equations (2)–(4), respectively. This allows the curve obtained under drained weight conditions to be utilized as parameter values for predicting the corresponding salinity conditions.

**Figure 10:**
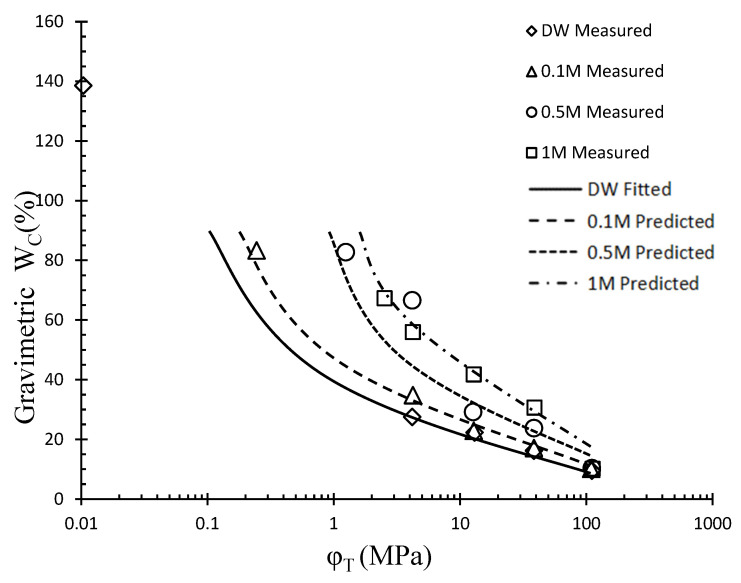
The Fredlund and Xing model verification considering the effect of salinity.

In contrast to classical models of the water retention characteristic, this model takes the influence of salinity into account on the WRC during the drying process. Initially, the parameter values corresponding to DW conditions are obtained by measuring the drying curve of GCL and fitting it using the Fredlund and Xing model. Subsequently, Equations (2)–(4) are utilized to determine the values of parameters ‘a’, ‘b’, and ‘c’ under different salinity levels. This allows the Fredlund and Xing model to predict the WRC of GCL under specific salinity conditions. To assess the effectiveness of the model prediction method, comparisons are made with the existing literature data [12]. The results, presented in Figure 10 and Table 4, indicate a strong agreement between the determined equation and the observed effects of salinity on the WRC of GCL hydrated with NaCl solution. Consequently, when the WRC under DW conditions has been established, this model enables the prediction of the influence of NaCl on the WRC.

## 4. Conclusions and Future Work

The primary objective of this study is to investigate the impact of salinity on the WRC of GCLs. The drying process of GCLs hydrated with various NaCl solution salinities is conducted under constant volume conditions using a dew point potentiometer. The following key findings are obtained:

1. GCL hydrated by NaCl solution with higher salinity has higher suction at the same water content. The suction increases with the decrease in water content on the drying path. To predict the WRC considering the salinity effect, the drying curve under distilled water (DW) conditions is used as a basis, and the WRC of GCLs under different salinities can be predicted using the Fredlund and Xing model along with the relationship between parameters and osmotic suction.
2. The salinity of the pore water of GCL hydrated by NaCl solution increases on the drying path until reaching a constant state. According to the electric double layer theory, the changing salinity alters the microstructure of clay. By keeping the drying process going, crystals will be precipitated in GCL and attached to the geotextile or bentonite components of GCL, which can be seen from SEM images. Consequently, the increase in total suction is not solely attributed to the osmotic suction, but also to the alteration of pore salinity, which impacts the pore structure of bentonite and alters the matric suction. The precipitation of NaCl crystals may contribute to the overall increase in total suction.

However, it should be noted that only one kind of GCL is used in this experiment and the complete GCL structure is taken as the experimental object, which inevitably has limitations. The use of a complete GCL structure makes it impossible for the study to have a large number of references, as the study of bentonite and geotextile may lead to differences. At the same time, different GCLs can obtain similar trends, which only need to do the same work and then summarize. GCLs, serving as anti-seepage barriers in landfills, are exposed to a more complex composition of salt solutions in their operational environment. Therefore, the influence of salinity, density, multiple ions, and other factors has not been considered, and the fitting parameter relationship observed in this experiment may not fully explain all the changes in GCL behavior. Further research is necessary to explore the relationship of the fitting parameters in the Fredlund and Xing model under the influence of various factors. This will enable more accurate predictions of the WRC on the drying path of GCLs under the influence of leachate in practical applications.

## Figures and Tables

**Table 1:** The properties of GCL.

Property	Value
Montmorillonite content	≥80 wt% (XRD)
Carbonate content	≤1–2 wt%
Particle size	Powdered (i.e., 80% passing 75 micron sieve)
Cation exchange capacity	≥70 meq/100 g
Free swell index	≥24 mL/2 g
Fluid Loss	≤18 mL
Mass per unit area, total GCL (@ 0% Moisture)	≥4200 g/m^2^
Thickness GCL, total (mm)	≥5.4 mm

**Table 2:** Classical models for water retention curve.

Number	Model	Notations	Researchers
1	$\ln\left(\varphi \right)=a+bln(\theta_{\omega })$	φ is the soil suction $\theta_{\omega }\;$is the water content *a*, *b* are curve fitting parameters	Williams [30]
2	$\theta_{\omega }=\theta_{r}+\frac{\theta_{s}-\theta_{r}}{1+a\varphi^{b}}$	φ is the soil suction $\theta_{\omega }\;$is the water content $\theta_{s}$ is the saturated water content $\theta_{r}$ is the residual water content *a*, *b* are curve fitting parameters	Gardner [31]
3	$\frac{\theta_{\omega }-\theta_{r}}{\theta_{s}-\theta_{r}}={\left(\frac{1}{1+a\varphi^{b}}\right)}^{c}$	φ is the soil suction $\theta_{\omega }\;$is the water content $\theta_{s}$ is the saturated water content $\theta_{r}$ is the residual water content *a*, *b*, *c* are curve fitting parameters	Van Genuchten [32]
4	$\theta_{\omega }=\frac{\theta_{s}}{{(\ln\left(e+{\left(\frac{a}{\varphi }\right)}^{b}\right))}^{c}}*$ $\left[1-\frac{\ln\left(1+\frac{\varphi }{\varphi_{r}}\right)}{\ln\left(1+\frac{1000000}{\varphi_{r}}\right)}\right]$	φ is the soil suction $\theta_{\omega }\;$is the water content $\theta_{s}$ is the saturated water content $\varphi_{r}\;$is the soil suction corresponding to residual water content *a*, *b*, *c* are curve fitting parameters	Fredlund and Xing [33]

**Table 3:** Parameters of WRC fitted by Fredlund and Xing model.

Salinity	Osmotic Suction (MPa)	a	b	c	R^2^
DW	0	1.482	3.291	0.843	0.992
0.1 M	0.462	1.603	2.499	0.913	0.990
0.2 M	0.915	2.232	1.977	0.939	0.991
0.5 M	2.281	3.035	1.894	1.034	0.999
2 M	9.78	11.032	1.700	1.255	0.933

**Table 4:** Parameters of Fredlund and Xing model verification.

Salinity	a	b	c	R^2^
DW	0.337	1.875	0.953	0.967
0.1 M	0.774	1.772	1.015	0.719
0.5 M	2.116	1.019	1.336	0.936
1 M	4.967	0.939	1.412	0.561

## Data Availability

All data that support the findings of this study are included within the article.
